# Multi-parametric cardiovascular magnetic resonance imaging detects subclinical myocardial involvement in patients diagnosed with phaeochromocytoma

**DOI:** 10.1186/1532-429X-17-S1-P271

**Published:** 2015-02-03

**Authors:** Vanessa M Ferreira, Mafalda Marcelino, Stefan K Piechnik, Claudia Marini, Theodoros D Karamitsos, Jane M Francis, Jayanth R Arnold, Radu Mihai, Julia D Thomas, Maria Herincs, Marta Korbonits, Zaki Hassan-Smith, Wiebke Arlt, Niki Karavitaki, Ashley Grossman, John Wass, Stefan Neubauer

**Affiliations:** Division of Cardiovascular Medicine, Radcliffe Department of Medicine, University of Oxford, Oxford, UK; Oxford Centre for Diabetes, Endocrinology & Metabolism (OCDEM), University of Oxford, Oxford, UK; Dept. of Surgery, Oxford University Hospitals NHS Trust (OUH), Oxford, UK; Dept. of Endocrinology, Barts and the London School of Medicine, Queen Mary University of London, London, UK; Centre for Endocrinology, Diabetes and Metabolism (CEDAM), University of Birmingham, Birmingham, UK

## Background

In patients with phaeochromocytoma, acute or chronic exposure to catecholamines may lead to cardiac pathology, including left ventricular (LV) hypertrophy, myocardial infarction, stress-induced cardiomyopathy and heart failure. The burden of myocardial involvement in this disease with systemic effects is unknown. In this prospective, multicentre study, we sought to describe the variety and incidence of cardiac abnormalities in patients diagnosed with phaeochromocytoma using multi-parametric cardiovascular magnetic resonance (CMR) imaging.

## Methods

We studied 50 patients diagnosed with phaeochromocytoma. Twenty patients (n=20, age 51±14 yrs) newly-diagnosed with confirmed phaeochromocytoma prospectively underwent CMR before and after curative surgical resection of the phaeochromocytoma (median follow-up 1 year). In addition, 30 patients (n=30, age 52±14 yrs) previously diagnosed with phaeochromocytoma who had curative surgery were also recruited for cardiac characterisation. Patients with known cardiac conditions were excluded. CMR included cine imaging for global and regional LV function, dark-blood T2-weighted imaging for oedema and late gadolinium enhancement imaging to detect the presence and patterns of any scarring.

## Results

In patients with newly-diagnosed phaeochromocytoma, the mean LV ejection fraction was 67±10% (EF range 47-88%); of these patients, 20% (n=4/20) had mild global LV dysfunction (EF 47-56%). A significant proportion (65%, n=13/20) demonstrated scarring, all with a non-ischaemic pattern, but these areas were small (<10% myocardium); no patient had evidence of myocardial infarction (isolated subendocardial scarring). One patient demonstrated global myocardial oedema with normal EF. All LV dysfunction or oedema were reversible and normalised at postoperative follow-up. In patients previously-diagnosed who already had had curative surgery, the mean LVEF was essentially normal (73±7%) with only one patient (3%) who had mild global LV dysfunction (EF=56%). Compared to the newly-diagnosed patients, a significantly smaller proportion of previously-diagnosed patients (17% vs. 65%; p<0.001) demonstrated areas of scarring, which were also small in areas with a non-ischaemic pattern, except for one patient who suffered a small myocardial infarction.

## Conclusions

Subclinical cardiac abnormalities are frequent findings on CMR in patients newly-diagnosed with phaeochromocytoma, including mild LV dysfunction, myocardial oedema and small areas of non-ischaemic scarring, with the former two demonstrating normalization after surgical resection of the phaeochromocytoma. In patients who had previously undergone curative surgical resection of their phaeochromocytomas, the incidence of cardiac abnormalities is lower, predominantly consisting of small areas of non-ischaemic fibrosis.

## Funding

VMF and SKP acknowledge funding from the National Institute for Health Research (NIHR) Oxford Biomedical Research Centre based at The Oxford University Hospitals NHS Trust and the University of Oxford.VMF received funding from the Alberta Innovates Health Solutions (AIHS) Clinical Fellowship and the University of Oxford Clarendon Fund Scholarship. SN acknowledges support from the British Heart Foundation Centre of Research Excellence, Oxford.

**Figure 1:**
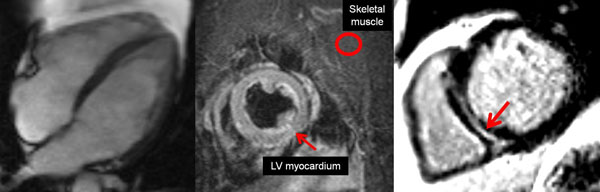
Cardiovascular magnetic resonance imaging techniques for myocardial tissue characterisation. (Left) cine movie imaging for left ventricular function (4-chamber view at end-diastole). (Middle) Oedema imaging using dark-blood T2-weighted CMR. Short-axis image of the ventricles. Note LV myocardial signal intensity compared to skeletal muscle signal intensity is >1.9, consistent with global myocardial oedema. (Right) Late gadolinium enhancement (LGE) imaging. Normal myocardium is black. Red arrow points to a mid-wall scar in the inter-ventricular septum, typical of non-ischaemic type scarring.

**Figure 2:**
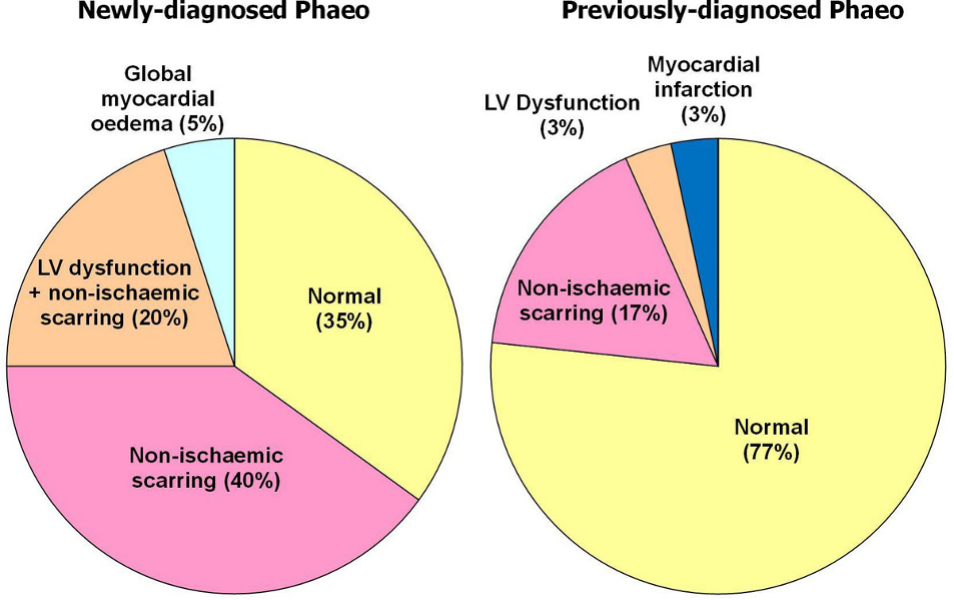
Cardiac abnormalities in patients with phaeochromocytoma as detected by cardiovascular magnetic resonance (CMR) using cine, T2-weighted and LGE imaging. (Left) Patients newly-diagnosed with phaeochromocytoma, before curative surgery. (Right) Patients previously diagnosed with phaeochromocytoma treated with curative surgery.

